# Kampala manifesto: Building community-based One Health approaches to disease surveillance and response—The Ebola Legacy—Lessons from a peer-led capacity-building initiative

**DOI:** 10.1371/journal.pntd.0006292

**Published:** 2018-04-02

**Authors:** Petra Dickmann, Andrew Kitua, Franklin Apfel, Nigel Lightfoot

**Affiliations:** 1 dickmann risk communication drc |, London, United Kingdom; 2 Jena University Hospital, Jena, Germany; 3 Southern Africa Centre for Infectious Disease Surveillance (SACIDS), Morogoro, Tanzania; 4 World Health Communication Associates (WHCA), Compton Bishop, United Kingdom; 5 Connecting Organizations for Regional Disease Surveillance (CORDS), Lyon, France; 6 Chatham House, Centre on Global Health Security, London, United Kingdom; Institute for Disease Modeling, UNITED STATES

## Abstract

**Overview:**

International activities to respond to the Ebola crisis in West Africa were mainly developed and focussed around the biomedical paradigm of Western health systems. This approach was often insensitive to societal perception, attitude, and behavioural determinants and clashed with community-based health traditions, narratives, and roles, e.g., of community health workers. In this peer-led capacity-building initiative, these deficiencies were identified and analysed. Innovative, more locally focussed, community-based solutions were articulated. The new approaches described put local people at the centre of all preparedness, response, and recovery strategies. This paradigm shift reframed the role of communities from victims to active managers of their response and reacknowledged the strength of community-based One Health. We conclude that strategies should aim at empowering, not just engaging, communities. Communities can improve short-term crisis management and build longer-term resilience and capacities that are much needed in the current global health climate.

**Background:**

The Ebola outbreak in West Africa, 2014–2016, was unprecedented in scale, extent, and duration. The international community was slow to step up its assistance in this global public health emergency and then faltered when its infection control management approaches clashed with West African realities [1]. Outbreak response evaluations have identified the need to better integrate social science intelligence [2], better collaborate with communities [3,4], more effectively draw on the strength of community health workers [5], and critically question the paradigm of Western health systems, which focus on imposing ‘evidence-based’ solutions that lack external validity in affected communities; i.e., they too often recommend actions that are inconsistent with, ignore, or violate traditional behaviours [6].

While there appears to be a consensus now on what needs to be done, how to achieve these goals remains a challenge.

## Ebola: Intensified preparedness programme

In order to identify practical ways to enhance the capacity of health workers and influencers to develop and articulate local and sustainable Ebola response, control, and recovery solutions for affected and not yet affected communities, an Ebola: Intensified Preparedness Programme (IPP) was set up in 2015. This initiative was funded by The Rockefeller Foundation, using its emergency funds, and managed by Connecting Organizations for Regional Disease Surveillance (CORDS). This approach worked in addition and alongside other initiatives, such as those by the United Nations (e.g., UNMEER) and other international organisations (i.e., International Federation of the Red Cross, UNICEF, World Health Organisation). These also included biomedical capacity support such as mobile laboratories by clinical research institutions, the United States Centers for Disease Control (CDC), and the German Robert Koch Institute, to name just a few. The coordination of various response capacity-building initiatives proved to be very challenging, and an International Interagency Ebola Communication Coordination Call (IIECCC) was set up that was chaired by the US CDC. The IPP contributed frequently to this interagency briefing and used other opportunities during meetings with specific agencies to share its approach and outcomes.

IPP brought together community-based health ‘shapers’ with specific experience from previous Ebola outbreaks in Uganda and DR Congo [7]. These ‘shapers’ included a mix of health professionals, community leaders, journalists, policy makers, religious leaders, and traditional healers, who influence the narrative of infectious disease management, risk communication, and community outreach in their local and national environments. A series of two-day training programmes to be held in different countries was designed for these ‘shapers’. They worked in facilitated small interactive groups using analytical matrix tools to elicit and capture insights on the underlying assumptions, perceptions, and beliefs of infection control management in their communities and countries and identify ways to shift the agenda and narrative to a more people-centred and community-based health paradigm.

The IPP analytic matrix tool was built on five action principles that were identified in a scoping meeting (in Dar es Salaam, Tanzania; 1–2 September 2014), with selected ‘shapers’ from Eastern and Central Africa with firsthand experience during Ebola outbreaks [7]:

- Community—work with communities, not against them;
- Communication—listen and gain insights into community assets and needs; develop respectful, culturally sensitive, two-directional risk-communication and awareness-raising approaches;
- Capacity building—build the capacity of local community leaders and local healthcare workers (lHCWs) in case detection and management;
- Coordination—build local multi-stakeholder networks, which coproduce action plans modifying/adapting a generic response plan for their specific Ebola preparedness contexts; and
- Culture—understand it as a key driver of community resilience and as a potential enabling or limiting factor to infection control.

Building on these principles, the IPP workshops were organised around a series of key questions:

Risk communication and risk management
  - Risk management—what specific considerations need to be taken on the local community level for infectious disease management in small-, medium-, and large-scale outbreaks?
  - Risk communication—how can we frame/shape/broaden communication to reflect different conceptions and perceptions of risks and respect the cultural drivers of communities?
  - How can we ensure that risk communication is not only about ‘giving health messages’ but also includes listening to and understanding local concepts and beliefs and works towards building relationships with communities to create supportive environments where people build their skills and make good decisions?
Infection control
  - What are the specific infection-control training needs at different levels of the community (e.g., healthcare workers [HCWs] on sub-district and district levels)? How can coordination with international organisations best be organised?
Collaboration
  - How can we strengthen generic response plans that build on existing infrastructures?
  - How can we encourage networking that effectively and efficiently shares information and expertise and develops sustainable collaborations between stakeholders?

This article reports on the outcomes of the first IPP held as a pilot training in Kampala.

## Results: Pilot training IPP Kampala

The pilot training in Uganda, Kampala (25–26 November 2014), resulted in a series of practical action insights and recommendations on ways to frame and promote the use of community-based outbreak response to bolster current approaches to infectious disease management and recovery on all levels. Findings are presented here as key recommendations and short video narratives by participants explaining the points made. The training was attended by participants from Uganda, Burundi, Zambia, Mali, DR Congo, Kenya, Zimbabwe, Ghana, and Tanzania.

## Training workshop insights, recommendations, and video narratives

### Change perception and confidence: From victims to active protagonists

The most striking insight was a shift in the way participants perceived and understood the scope of their roles as protagonists, champions, and leaders of community-based infection control management. At the beginning of the workshop, participants described the situation of Ebola preparedness, response, and recovery in ‘don’t have’ narratives. These narratives emphasised and bemoaned problems resulting from deficits in resources, the lack of scientific understanding of the disease in the population, and poor communication between health professionals, international aid workers, and communities. During the interactive work in small working groups, this perspective shifted into new powerful action narratives. These emphasise active whole-of-community ownership, leadership, and management of health, healthcare, and supply system logistic approaches to ongoing infectious disease challenges [8]. In articulating these new approaches, participants drew on evidence from previous Ebola outbreaks and their firsthand experience and stressed the pivotal but poorly acknowledged role that communities play in limiting the spread of and controlling the disease [8].

### Shift the focus of community engagement: From telling to listening, from formal leaders to informal leadership

Traditionally, ‘community engagement’ approaches are often limited to better explaining to local people what they have to do and how to better apply infection control recommendations. Participants elucidated mismatches with this conventional top-down approach to infection control, which they felt did not truly acknowledge the resourcefulness and strength of communities and too often missed critical action-enhancing insights [9].

Participants pointed to and analysed examples of information sharing and communication that was too focussed on ‘official’ scientific information and ‘official’ community leaders and ignored informal communication and leadership systems of communities (e.g., ‘rumours’ and ‘Queen Mums’). International recommendations and behaviour advice were seen as frequently inadequate, insensitive, and patronising; adequate explanations were often missing and recommendations ignored the critical identity-building social and religious forces of societies [10].

Participants argued for more inclusive collaboration and participatory actions that eliminate the barriers between ‘we’ and ‘they’. They advocated for a better mutual understanding and the need for negotiating valid compromises to limit the spread and control the disease. They pointed out, for example, that communities are able to modify their cultural or religious practices to reduce risk and create safer environments, but these modifications have to be framed in ways that take account of local reasoning and what matters for both communities and infection control [11].

### Shift power to the people

Participants criticised the international development and deployment model as cementing dependency on international aid. While acknowledging that there are huge capacity-building needs and demands in regard to education, training, and professional development, participants stressed that these have to be built on local terms, assets, needs, and conditions. International aid is too often designed to please the ‘senders’ but does not meet the local needs. They saw a discrepancy in the priority setting of the response; while the international community engaged along the scientific medical rationale in vaccine production and clinical trials, participants stressed the important but underestimated role of psychosocial support needs [12].

### Realising the ‘beauty’ of the Ebola legacy: Community-based One Health

Participants suggested that response, control, and recovery all needed a much broader approach, in which health is only one of the relevant sectors for improvement.

They called for a strengthening of community-based One Health approaches that include sectors such as trade, travel, research, etc. The ‘beauty’ of Ebola’s legacy is seen as creating a unique opportunity to build and strengthen health and, importantly, local health supply systems according to local needs and wishes. Limiting or terminating the dependence on international aid would require strengthening local resources. This would require supporting local economies to develop local solutions. However, as sectors are still underdeveloped, they need to join forces with other disciplines and sectors on all levels and collaborate. Participants envisioned organising regional meetings with local stakeholders to build networks (research and laboratory), engage with industry, and create business fora for innovation (e.g., rapid tests) [13].

## Conclusion

Participants suggested a much broader and more creative approach to response management and recovery. They engaged in socioeconomic and political thinking about ownership, confidence, and independence that could support both the short-term crisis management and longer-term resilience and capacity building in the region. They called for a Kampala manifesto that shifts power to the people and promotes a community-based One Health approach.

### Rollout of the pilot

Trainees of this pilot training joined CORDS staff in holding further IPP trainings throughout 2015 in affected and not yet affected countries in Africa. In total, over 100 multipliers and trainers were directly involved. Training delivered by pilot trainees and other in-country trainers were held:

- 13–14 January 2015—Accra, Ghana, with participants from Guinea, Ghana, and Burkina Faso;
- 3–4 February 2015—Nairobi, Kenya, with participants from South Sudan, Kenya, Burundi, Tanzania, and Malawi;
- 17–18 February 2015—Conakry, Guinea, with participants from Guinea, Liberia, Sierra Leone, Togo, and Senegal; bilingual (English–French);
- 3–4 March 2015—Abuja, Nigeria, with participants from Nigeria, Tanzania, Ghana, Serbia, and Cameroon; and
- 24–25 March 2015—Bamako, Mali, with participants from Mali, DR Congo, Cameroon, Burundi, Mali, Senegal, and Côte d’Ivoire (French course).

## References

[1] Petherick A. Ebola in west Africa: learning the lessons. Lancet. 2015;385(9968):591–2. doi: 10.1016/S0140-6736(15)60075-7 25682526 10.1016/S0140-6736(15)60075-7

[2] Abramowitz SA, Bardosh KL, Leach M, Hewlett B, Nichter M, Nguyen VK. Social science intelligence in the global Ebola response. Lancet. 2015;385(9965):330 doi: 10.1016/S0140-6736(15)60119-2 25706852 10.1016/S0140-6736(15)60119-2

[3] Kieny MP, Dovlo D. Beyond Ebola: a new agenda for resilient health systems. Lancet. 2015;385(9963):91–2. doi: 10.1016/S0140-6736(14)62479-X 25706456 10.1016/S0140-6736(14)62479-X PMC5513100

[4] Kutalek R, Wang S, Fallah M, Wesseh CS, Gilbert J. Ebola interventions: listen to communities. Lancet Glob Health. 2015;3(3):e131 doi: 10.1016/S2214-109X(15)70010-0 25618243 10.1016/S2214-109X(15)70010-0

[5] The Lancet Global Health. Community health workers: emerging from the shadows? Editorial. The Lancet Global Health. 2017;5(5): e467.7. 10.1016/S2214-109X(17)30152-3 28395831

[6] Chandler C, Fairhead J, Kelly A, Leach M, Martineau F, Mokuwa E, et al Ebola: limitations of correcting misinformation. Lancet. 2015;385(9975):1275–77. doi: 10.1016/S0140-6736(14)62382-5 25534188 10.1016/S0140-6736(14)62382-5

[7] Dickmann P, Kitua A, Kaczmarek P, Lutwama J, Masumu J, Karimuribo E, et al Lessons learned from previous Ebola outbreaks to inform current risk management. Emerg Inf Dis. 2015;21(5). doi: 10.3201/eid2105.142016

[8] Participants' statement during the Kampala pilot training [cited 2018 Mar 19]. Available from: https://www.youtube.com/watch?v=phx5UYlhiw8

[9] Participants' statement during the Kampala pilot training [cited 2018 Mar 19]. Available from: https://www.youtube.com/watch?v=zIHcvNqS-1s

[10] Participants’ statement during the Kampala pilot training [cited 2018 Mar 19]. Available from: https://www.youtube.com/watch?v=Pv_pgO59lDo

[11] Participants’ statement during the Kampala pilot training [cited 2018 Mar 19]. Available from: https://www.youtube.com/watch?v=Pe3S8TdY598

[12] Participants’ statement during the Kampala pilot training [cited 2018 Mar 19]. Available from: https://www.youtube.com/watch?v=-jDUh4EanWw

[13] Participants’ statement during the Kampala pilot training [cited 2018 Mar 19]. Available from: https://www.youtube.com/watch?v=kJJwQ7ua4WQ

